# Selective Class-Aware Refinement (SCAR) Method for Microrobot Detection in Ultrasound Images

**DOI:** 10.3390/s26185798

**Published:** 2026-09-13

**Authors:** Ahmed Almaghthawi, Changyan He, Suhuai Luo, Furqan Alam, Saqib Qamar, Lingbo Cheng

**Affiliations:** 1 School of Computer and Information Sciences, University of Newcastle, Newcastle 2308, Australia; suhuai.luo@newcastle.edu.au; 2 School of Engineering, University of Newcastle, Newcastle 2308, Australia; lingbo.cheng@newcastle.edu.au; 3 Department of Computer Science, College of Applied Sciences, King Khalid University, Muhayil 63772, Saudi Arabia; 4 Faculty of Computing and Information Technology (FoCIT), Sohar University, Sohar 311, Oman; falam@su.edu.om; 5 Department of Intelligent Systems, KTH Royal Institute of Technology, 10044 Stockholm, Sweden; sqama@kth.se

**Keywords:** healthcare, real-time detection, ultrasound imaging, microrobots, YOLO detectors

## Abstract

Microrobots have the power to transform healthcare and the medical sector by improving diagnosis and targeted drug delivery. Real-time detection of microrobots is critical to ensure reliable operation. Ultrasound (US) imaging is used for detection because it is non-ionizing, low-cost, and easy to set up. Using US images is challenging because certain microrobot shapes (classes) remain difficult to detect due to their small size, low contrast, and unstable appearance. This paper proposes Selective Class-Aware Refinement (SCAR), a lightweight post-detection method built on a baseline YOLO detector that boosts its real-time detection capabilities. SCAR improves weak-class detection for visually challenging objects that are small, low-contrast, or unstable, without sacrificing inference speed. To identify weak classes, SCAR is designed to analyze class-specific detection behavior offline. It does so by examining failure statistics and visual difficulty cues. During prediction, SCAR selectively focuses on difficult-to-predict classes using a compact specialist detector applied to adaptive local crops whose size scales with the detected object’s dimensions. SCAR limits the refinement to small local regions rather than reprocessing the entire US frame. SCAR preserves near-baseline frame-per-second (FPS) while improving detection. We conducted experiments on the open dataset USMicroMagSet and tested the method on two YOLO baselines and five other state-of-the-art detectors. The results show that SCAR improves weak-class performance with limited computational overhead: it raises weak-class detection accuracy from near-zero to a usable level, improves overall mAP, and maintains consistent performance.

## 1. Introduction

Microrobots are emerging as a technological paradigm with the potential to transform modern biomedical engineering, leading to significant improvements in diagnostics and treatments. These tiny robots can operate inside confined biological environments that conventional surgical tools cannot easily reach [1]. They have many applications in the medical and healthcare sectors, including minimally invasive interventions, targeted drug delivery, localized sensing, and precision therapeutic procedures [2,3,4]. One major hurdle to the clinical deployment of microrobots is the need for continuous visual monitoring within the human body, which remains to be achieved. Accurate and robust tracking is essential for safe and reliable operation of microrobots.

**Figure 1:**
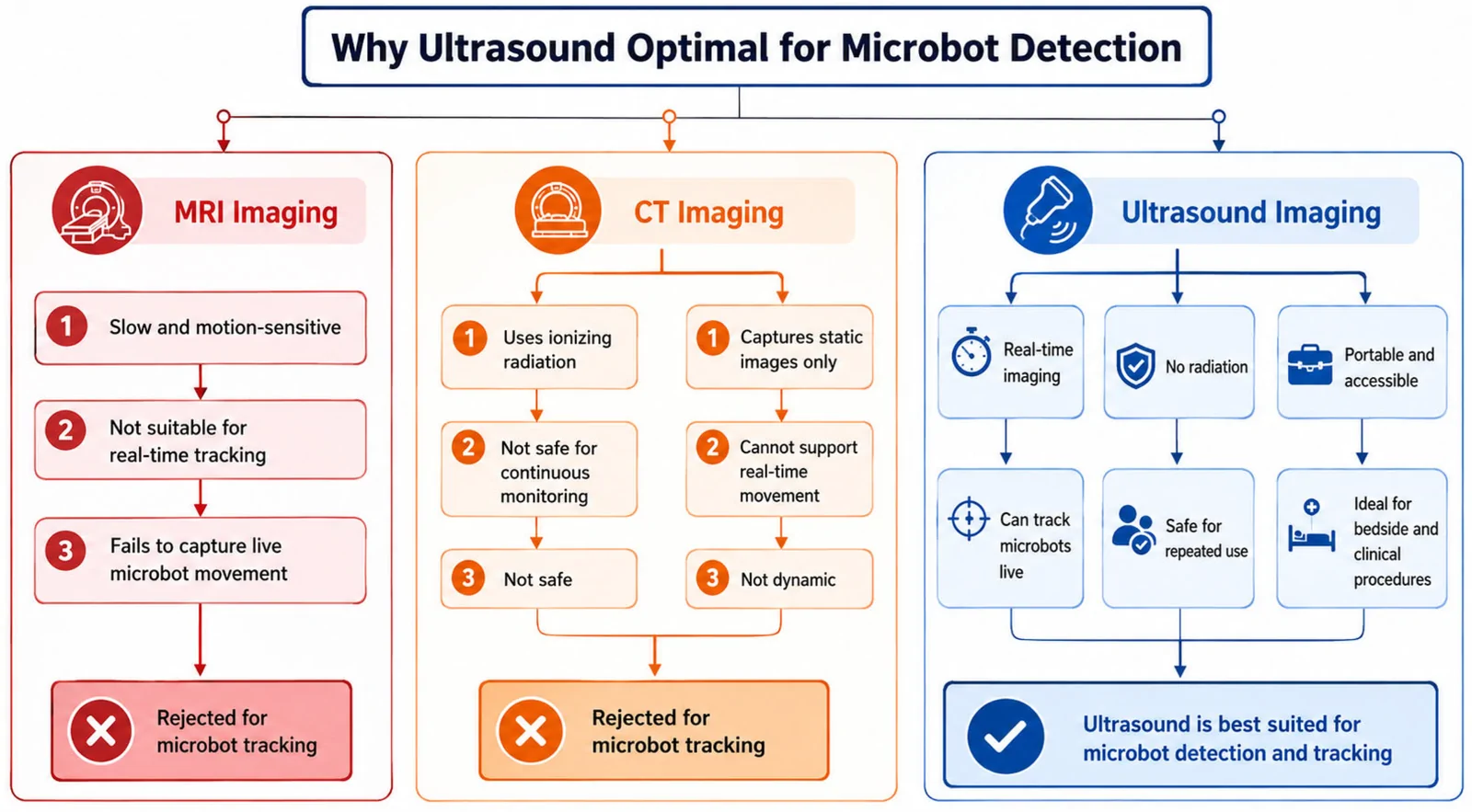
Block diagram showing why ultrasound (US) imaging is the optimal choice for microrobot detection in real-time.

Among imaging modalities, ultrasound (US) is favored for microrobot monitoring because it is radiation-free, non-invasive, real-time, has high temporal resolution, and is low-cost. Comparatively, other imaging modalities, such as magnetic resonance imaging (MRI) and computed tomography (CT), are costly and can pose safety risks. Those approaches can lead to severe health issues, particularly in continuous microrobot scenarios [5,6]. The US is highly suitable, as depicted in Figure 1, for continuous and repeated monitoring during microrobot-assisted procedures. Nevertheless, US images pose substantial challenges for microrobot detection because they have lower spatial resolution and contain speckle noise, low contrast, shadowing artifacts, and blurred boundaries. Further, microrobots are very small, which increases their visual similarity to surrounding structures.

Recent advances in computer vision have significantly improved the accuracy of object detection in medical imaging tasks. However, microrobot detection methods in US images are in their infancy. For the microrobot detection problem, two main aspects must be investigated: reliability and higher detection accuracy. In this paper, we address the above by achieving (1) lightweight detection models and (2) real-time detection. A few deep learning-based detector architectures have demonstrated promising results in identifying complex visual patterns of microrobots in US images [7]. However, a major research gap, as highlighted in [7] is that common architectures such as ConvNeXt, Res2NeXt-101, and ResNeSt-269 achieve high detection accuracy but are computationally impractical for deployment in real-world clinical settings.

In contrast, YOLO-based models show real-time detection capabilities [7]. However, they fail in difficult-to-detect microrobot classes, which are classes that either look similar to the local or global environment.

Existing solutions either redesign the detector architecture by adding feature-extraction modules to create a hybrid model or retrain larger models. The obvious drawback is that complexity and parameter count increase, further raising computational requirements and inference time. Thus, it is not suitable for real-time US applications. To address this limitation, we propose the Selective Class-Aware Refinement (SCAR) method, which achieves higher detection accuracy and is suitable for real-time deployment in clinical settings.

### 1.1. Aims and Contributions

This work aims to develop a method to improve the accuracy and reliability of microrobot detection in US images, suitable for real-time medical imaging applications. The contributions of this work are as follows:

- Analyze class-wise detection performance and identify where state-of-the-art detectors face challenges and where their performance degrades.
- Design a selection mechanism that identifies the hard-to-detect class from the baseline’s error statistics.
- Apply a gated mechanism that rejects classes for which refinement cannot help, ensuring that refinement executes only on the selected class.
- Evaluate the proposed method by measuring detection accuracy, computational cost, and throughput on high-end and low-end GPUs, and report how much the results vary over repeated runs.

### 1.2. Structure of the Paper 

This paper consists of six sections that explore deep learning-based detectors for microrobot detection in US. In Section 2, we conducted a literature review analyzing the role of US imaging and existing work in this field. Section 3 explains the proposed method in detail. It includes the experimental setup and datasets. Section 4 discusses the results comprehensively, while Section 5 presents an ablation study in which we critically analyze the components of the proposed method to assess their impact on performance. Finally, Section 6 concludes the paper.

## 2. Literature Review

Real-time microrobot detection remains a significant challenge in medical applications, including targeted drug delivery, minimally invasive surgical procedures, and localized diagnostic interventions [8,9,10]. Furthermore, microrobot tracking success is closely linked to detector performance, and ultrasound imaging provides important visual feedback during medical interventions [11]. US imaging has significant issues with spatial resolution and signal-to-noise ratio, which can be a major challenge for microrobot detection in complex operating environment inside the human body [8,9,10,11,12]. Various medical imaging techniques, such as MRI, CT, optical imaging, and ultrasound, provide valuable support in developing machine learning and deep learning approaches for microrobot detection and tracking [13,14,15]. MRI can visualize soft tissues and vascular structures. Tong et al. [16] show in their study how multimodality imaging can identify breast tumors using a US contrast agent, which enhances tumor visualization and provides information that informs surgical decisions and treatment plans.

Furthermore, CT scans provide rapid imaging and high spatial resolution, making them well-suited for trauma-related applications and for assessing complex anatomical structures. Their effectiveness in scoliosis evaluation is particularly significant, as they provide detailed visualization of vertebral rotation, as does US imaging [17]. MRI and CT scans have a major disadvantage compared to US, as shown in Figure 1, and are not suitable for microrobot detection and tracking. Additionally, MRI systems are complex and costly to deploy and maintain in traditional clinical settings [18,19]. CT scan systems can damage human cells [20,21].

In contrast, US imaging does not damage human cells and has low deployment and maintenance costs, making it an optimal candidate for detecting and tracking microrobots in clinical settings. Numerous studies have shown that US imaging is well suited for real-time monitoring of microrobots [11] and for clinical diagnosis [22,23,24]. It is a far more suitable candidate than MRI and CT scans for point-of-care use because it is non-ionizing, cost-effective, has high temporal resolution, and is easily deployable, maintainable, portable, and compact, as shown in Figure 1. Figure 1 also highlights additional reasons why US is the optimal choice for microrobot detection and tracking, along with some real challenges. First, noise is naturally present in US imaging. Second, US has low contrast and limited resolution. This adds a new challenge to microrobot detection and tracking methods. Today, AI has advanced, and particularly the computer vision field has taken great strides with more complex and technologically sophisticated algorithms, which provide greater accuracy. In parallel, initial work on detecting and tracking microrobots using US has begun.

One of the most widely used microrobot detection methods in US imaging for various medical applications is ConvNet-based methods [9,25]. In this initiative, Botros et al. [9] proposed a method that produced around 95% detection and 93% tracking accuracy. Further, Sadak [25] introduces an explainable CNN model that uses an Attention-Fused Bottleneck Module (AFBM). The AFBM improves microrobot classification and localization using US imaging. The proposed method also outperforms YOLO-based models. The reported maximum mAP and IoU are 0.909 and 0.95, respectively. Li et al. [10] proposed a method focused on YOLOv5 and introduced a new variant of it for microrobots ranging from 1 to 3 mm in size. It achieved 91%+ prediction accuracy and works for both 2D and 3D detection and tracking in vitro. 

Reinforcement learning (RL) moved far beyond its initial application of game playing to robotics, autonomous systems, recommendation, finance, healthcare, and even LLMs. Similarly, vision-based transformers (ViTs) moved beyond their ImageNet classification application to object detection, segmentation, and medical imaging. RL [26], along with ViTs [27,28], is also emerging as a strong contender for microrobot detection methods. Today, most R&D is concentrated on diagnosis-based detection, not microrobot detection. In their study, Medany et al. [29] focused on controlling and detecting microrobots using US imaging. They introduced an approach that uses image feedback to support real-time operation in complex environments with limited training data. The proposed method achieved around 90% accuracy across multiple scenarios. Liu et al. [28] developed a method for real-time detection of robotic capsules using 3D US, fusing CNNs and transformers. This method exploits transformers’ ability to capture long-range context, improving detection accuracy. The proposed system produced over 90% detection accuracy with a localization error of 1.5 mm. However, microrobots face manual navigation challenges. This is due to complex dynamics and multiple motion parameters. Schrage et al. [30] use a large dataset of 100 K images to train an RL-based autonomous navigation method for accurate detection and control of guided microswarms using primary and secondary acoustic radiation forces, enhancing robustness and adaptability. 

In the human body, the gastrointestinal tract is complex because of its complex internal anatomy. This makes microrobot detection difficult because it requires tracking capsule location and operational state. To address this problem, Liu et al. [31] proposed a deep learning-based method to enhance intelligent robotic behaviors. This method fuses hierarchical attention with US imaging for both state classification and 2D in-plane pose estimation of capsule robots. The proposed method achieved 97% accuracy for mechanism state detection with 0.24 mm mean centroid error and orientation error. One challenge in microrobot detection is pairing high accuracy with high Frames Per Second (FPS), which YOLO-based detectors deliver, making them an ideal choice for real-time clinical deployment.

Some studies have also introduced refinement-based detection, such as Cascade R-CNN [32], a multi-stage architecture that uses a sequence of detectors trained with increasing IoU thresholds, with the same cascade procedure applied at inference. RefineDet [33], by contrast, consists of an anchor refinement module and an object detection module: the first filters out negative anchors and coarsely adjusts their locations and sizes, and the second takes the refined anchors as input. Another such study, Grid R-CNN [34], adopts a grid-guided localization mechanism and replaces box offset regression with grid point estimation to obtain high-quality localization. However, in all these methods, the refinement stage is part of the architecture and is applied to every proposal or anchor. They also aim to improve the quality of the detections the baseline already produces. Whereas SCAR derives the class to refine from the baseline’s error statistics and applies a gate that rejects classes where refinement cannot help. Therefore, refinement is invoked only where needed.

## 3. Methodology

This paper introduces a detection-refinement method, named Selective Class-Aware Refinement (SCAR), designed to improve microrobot detection in US images. The method improves accuracy while enabling a real-time, deployable approach for practical applications in routine clinical settings. The fundamental idea behind SCAR is to fuse (1) what already performs best and (2) develop only what is necessary to solve specific detection challenges. This fusion must be efficient and improve detection accuracy. First, a baseline YOLO detector is used to detect various microrobot classes with diverse shapes, sizes, and orientations in ultrasound imaging. The universal advantage of YOLO-based architecture is its real-time detection capabilities, making it a perfect fit for on-site applications. However, as Almaghthawi et al. [7] highlight, it relies heavily on illumination while neglecting texture. SCAR includes a refinement component that fuses offline, class-level diagnostic analysis with localized adaptive refinement during microrobot detection. A major challenge for SCAR is addressing YOLO’s architectural problems without sacrificing detection speed or frames per second (FPS), which is critical when moving from theoretical methods to real-world deployable ones. Section 3.2 explains it in detail. We measure performance using standard detection metrics and computational efficiency using FPS.

### 3.1. Dataset and Experimental Setup

**Figure 2:**
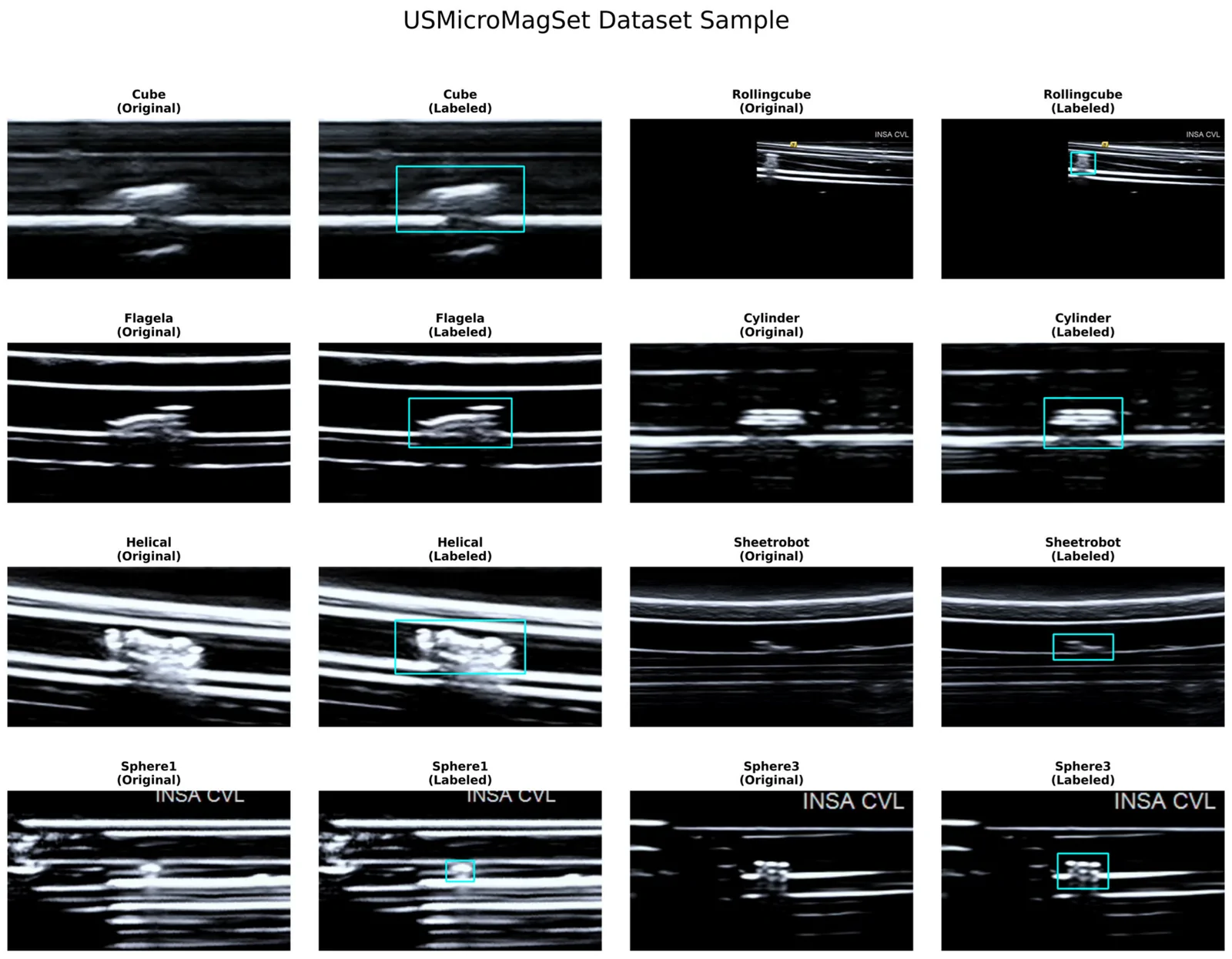
Sample of each microrobot ultrasound frame with unlabeled and labeled images with bounding boxes.

A major challenge in developing microrobot detection methods is the lack of availability of large and diverse training datasets. The USMicroMagSet [8] dataset is one of the few publicly available datasets available in its entirety and is used in this work and widely used for training and testing detection and tracking algorithms. Other datasets are private to the best of our knowledge. USMicroMagSet is a B-mode US dataset for microrobots available on IEEE DataPort, consisting of over 40,000 annotated US frames of size 1920 × 1080 pixels, of which 32,511 are usable. As depicted in Figure 2, it consists of eight different magnetic microrobot shapes with two locomotion modes. We used the exact folder structure provided in IEEE DataPort for the USMicroMagSet dataset to enable generalization and ensure the proposed method supports future work with fair comparative benchmarking. The dataset is split into 21,127 instances for training and 11,384 instances for testing across the eight microrobot classes. The training data merges the train and val folders; US images and test images are in the test folder. Furthermore, Figure 2 visualizes samples of unlabeled and labeled US images from USMicroMagSet.

We have used RunPod.io for all experimentation, including training, testing, and visualization. It is a commercial GPU cloud service platform. We used NVIDIA high-end RTX PRO (6000, 6000 WK) GPUs, built on the Blackwell architecture and delivering extreme throughput for next-generation AI workloads, and low-end RTX PRO 4000 GPUs for training and testing detection models. The dataset includes 1920 × 1080 images, which requires robust processing and computational infrastructure. The implementation uses the Ultralytics and PyTorch Vision libraries in a Python environment. We have standardized the core hyperparameters across the baseline and the proposed model.

### 3.2. Proposed Method

**Figure 3:**
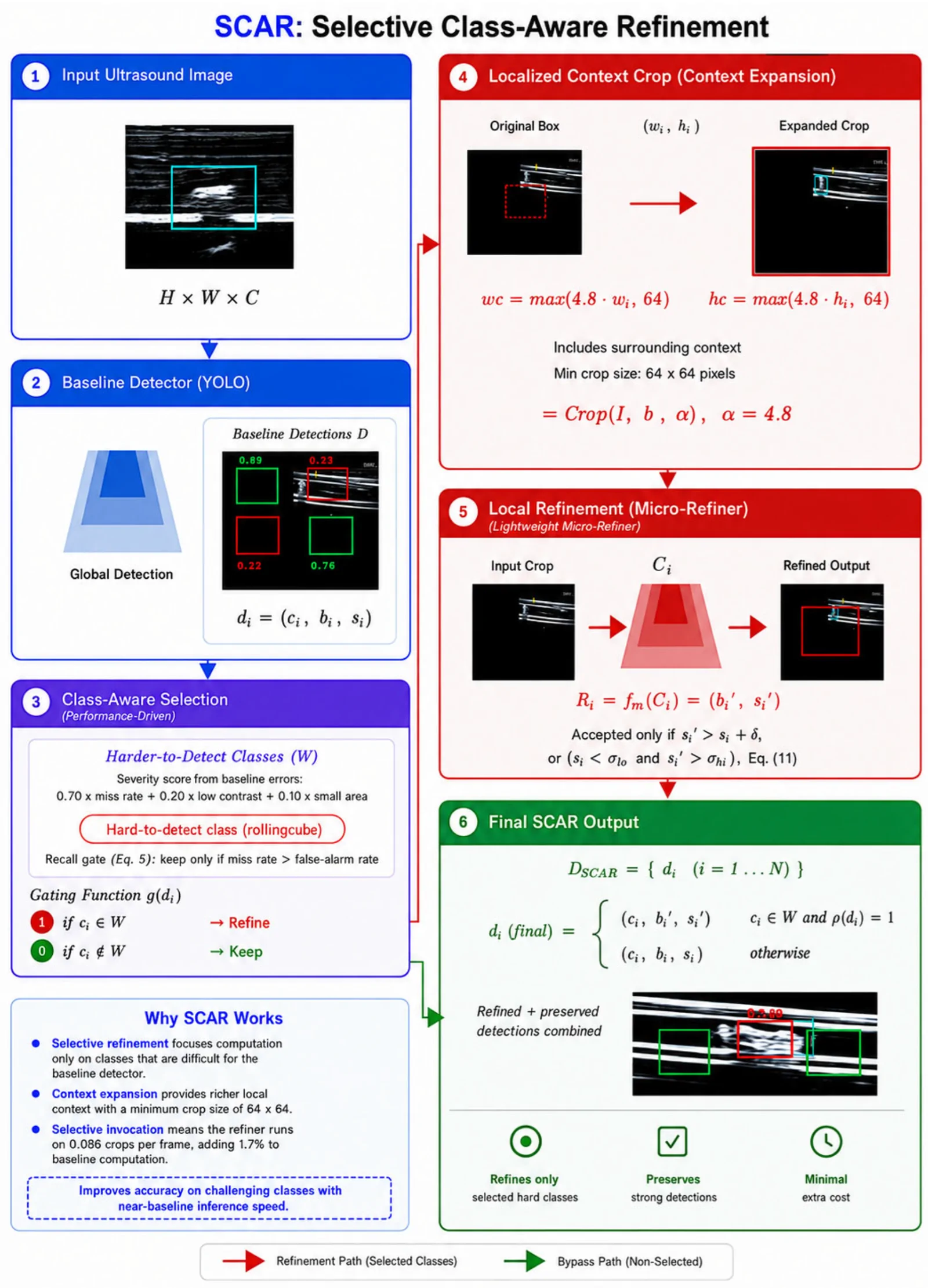
Self-explanatory block diagram of SCAR.

The SCAR method, as depicted in Figure 3, starts with an input US image, I, as a tensor, as defined in Equation (1). The image is processed by the baseline YOLO detector and generates a set of detections D, as expressed in Equation (2). As defined in Equation (3), each detection $d_{i}$ consists of a predicted class label, bounding box, and confidence score. Next, the SCAR task identifies difficult-to-detect classes, determined offline using baseline performance metrics as in Equation (4), and represented by the set W. The next step in SCAR is to use a class-aware gating function, which is applied to each detection as given in Equation (5) and determines whether a detection should be refined or preserved. The detections in set W are selected for further processing, while the others are bypassed for refinement.

Now, for the selected detections, SCAR uses contextual crop extraction. A local region has been extracted centered on the detected object using Equation (6). Here, the crop is expanded by a factor (α = 4.8), which controls how much surrounding context is included around the detected object. Rather than setting this factor manually, we select it via an offline Bayesian optimization procedure that searches for the value that maximizes overall validation performance. This guarantees sufficient surrounding information without increasing inference cost. Equations (7) and (8) compute the crop dimensions, ensuring a minimum size of 64 × 64 pixels while preserving the surrounding local context. The micro-refiner then processes the cropped region as in Equation (9), producing a refined bounding box and an updated confidence score that enable localized enhancement in a computationally efficient manner without reprocessing the entire image. For each selected weak-class detection, the refined output replaces the corresponding baseline detection, while non-selected detections remain unchanged. Lastly, as given in Equation (12), the SCAR output is constructed by combining refined and preserved detections, with the selected class set W updated using the refined values. The remaining detections remain unchanged. This results in better detection accuracy and lower computational cost. Further, Figure 3 presents a stepwise visualization of SCAR for ease of understanding.

The mathematical formulation of SCAR is given below:

#### 3.2.1. Baseline Detection

Let us assume that the input US image is expressed as

(1) $$I\in R^{H\times W\times C}$$

The baseline detector YOLO produces

(2) $$D_{b}=\{d_{i}{\}}_{i=1}^{N}$$

where each detection is expressed as

(3) $$d_{i}=\left(c_{i},b_{i},s_{i}\right)$$

where predicted classes, bounding boxes, and confidence scores are denoted by $c_{i},b_{i}$ and $s_{i}$, respectively.

#### 3.2.2. Class-Aware Selection

The harder-to-detect classes are identified offline using baseline detection evaluation benchmarks as

(4) W={c}

where c belongs to selected refinement classes.

We apply the gating rule, which is mathematically given as

(5) $$g\left(d_{i}\right)=\left\{\begin{array}{c} 1,\;c_{i}\in W\;\\ 0,\;c_{i}\notin W \end{array}\right.$$

where $g\left(d_{i}\right)=1$ denotes that refinement is activated and $g\left(d_{i}\right)=0$ denotes that the baseline is preserved.

The selection procedure is dataset-relative rather than a fixed class list. For each class, we evaluate the baseline on the training split to obtain a miss rate, a false-alarm rate, and the median object area and Weber contrast. A severity score weights the miss rate at 0.70 and the low-contrast and small-area priors at 0.20 and 0.10, respectively, so that classes failing through missed detections rank highest. Because the area and contrast terms are normalized against percentiles of the dataset itself, no absolute thresholds are carried over between datasets. The two highest-scoring classes are then passed through the gate of Equation (5), which retains only classes whose miss rate exceeds their false-alarm rate, since local refinement proposes additional candidates and cannot correct a class that already over-fires.

Table 1 reports the resulting statistics for both baselines. The rollingcube class ranks first under both detectors, with a miss rate 5.5 and 6.4 times higher than that of the next class, and it is the only class in the dataset whose miss rate exceeds its false-alarm rate under either detector. The complete ranking of all eight classes is identical for the two baselines despite their different architectures, indicating that the selection reflects a property of the data rather than of a particular detector.

#### 3.2.3. Contextual Crop Extraction

For the cases of selected detections,

(6) $$C_{i}=Crop\left(I,b_{i},\alpha \right)$$

where α=4.8

The Crop dimensions are given as

(7) $$w_{c}=max\left(4.8w_{i},64\right)$$

(8) $$h_{c}=max\left(4.8h_{i},64\right)$$

where the original detected object width, original detected object height, crop width, and crop height are denoted by $w_{i},\;h_{i},\;w_{c}$ and $h_{c}$, respectively.

Beyond the detected object, the formulations in Equations (7) and (8) help enlarge the cropped region to include local visual context. Simultaneously, we enforce a minimum crop size of 64 × 64 pixels. This ensures stable refinement for very small targets with limited computational overhead, preserving fast detection speed.

We select the expansion factor via Bayesian optimization using a Tree-structured Parzen Estimator with a fixed seed. The search range is the continuous interval α in [1.0, 5.0], with the upper bound set a priori so the crop remains local rather than approaching the full frame. The objective is the mAP(50–95) of the hard-to-detect class measured on a held-out validation split comprising 15% of the training images; we do not access the test set during the search. We ran nine trials per baseline, each executing the full matched chain of patch construction, 30 epochs of refiner training, refined inference, and evaluation, so the crop context is identical at training and inference time. The search required approximately 8 hours per baseline on a single NVIDIA RTX PRO 6000 GPU and is performed once offline, adding no inference cost. Both baselines independently selected α = 4.80.

#### 3.2.4. Local Refinement 

Now the micro-refiner will process the cropped region given as

(9) $$R_{i}=f_{m}\left(C_{i}\right)=\left(b_{i}^{\prime },s_{i}^{\prime }\right)$$

where the micro-refiner is denoted by $f_{m}\left(.\right)$, local crop by $C_{i}$, refined the bounding box by $b_{i}^{\prime }$, and refined the confidence score by $s_{i}^{\prime }$, respectively.

The micro-refiner is a hard-to-detect class detector that uses the same backbone and neck as the baseline detector it accompanies, with the detection head reduced from the eight dataset classes to a single output class representing the hard-to-detect category. It is initialized from the baseline checkpoint. This keeps the low-level US representations learned by the baseline and retrains only the decision layers. It does not classify among the eight microrobot categories; it inherits the class label from the baseline detection, as in Equation (12). Table 2 summarizes layer counts, parameter counts, training settings, and computational cost.

The refiner is trained only on cropped patches from the training split; the test split is not accessed at any stage of refiner construction. Positive patches are drawn from hard-to-detect class ground-truth instances that are both small and low-contrast, as given in Equation (10).

(10) $$H=\{g:\frac{w_{g}h_{g}}{WH}<\tau_{a}\;\wedge \;\kappa \left(g\right)<\tau_{c}\}$$

where $\tau_{a}$ and $\tau_{c}$ are the 25th percentiles of normalized object area and of Weber contrast over the training split, and $\kappa \left(g\right)$ is the contrast of instance g against a surrounding background ring. Negative patches are taken from regions where the baseline produced false positives on the training split, balanced one-to-one against positives, and discarded if they overlap a true hard-to-detect class instance. Patches are cropped using the same context expansion of Equations (7) and (8) that is applied at inference so that the training and inference distributions coincide. We re-normalize annotations to patch coordinates and map them to the single hard-to-detect class. Patch generation is seeded and therefore reproducible.

The refiner is trained for 30 epochs at an input resolution of 320 × 320 with a batch size of 128, mixed-precision arithmetic, and a fixed seed of 42. Rectangular training preserves the patch aspect ratio, and mosaic augmentation is disabled for the final 10 epochs. The optimizer is AdamW, automatically configured with a learning rate of 0.000833 and a momentum of 0.9, together with a final learning-rate factor of 0.01, a weight decay of 0.0005, and a three-epoch warm-up. The loss weights are 7.5 for the box term, 0.5 for the classification term, and 1.5 for the distribution focal term. Augmentation follows the baseline recipe, excluding vertical flip, rotation, shear, and perspective transforms, since ultrasound frames have a fixed acquisition geometry. We use identical settings for both baselines.

For a selected hard-to-detect class, detection with confidence $s_{i}$, the refiner output $\left(b_{i}^{\prime },\;s_{i}^{\prime }\right)$ replaces the baseline result only when it is decisively better, as given in Equation (11).

(11) $$\rho \left(d_{i}\right)=\left\{\begin{array}{c} 1\;\mathrm{if}\;s_{i}^{\prime }>s_{i}+\delta \vee (s_{i}<\sigma_{lo}\wedge s_{i}^{\prime }>\sigma_{hi})\\ 0\;\mathrm{otherwise}\; \end{array}\right.$$

with the fixed constants δ = 0.1, $\sigma_{lo}$= 0.3, and $\sigma_{hi}$ = 0.5. The first condition admits a refinement that improves confidence by a clear margin. At the same time, the second recovers instances that the baseline scored too low to report but that the refiner identifies with high confidence. When $\rho \left(d_{i}\right)$ = 0, the baseline detection is retained, so refinement cannot remove a detection that the baseline would have reported.

In terms of computational complexity, the refiner has approximately the same capacity as the baseline detector, and its efficiency comes not from reduced model size but from selective invocation. Because refinement is restricted to a single class and applied to local crops, the refiner is not executed on most frames. Measured over the full test set of 11,384 frames, it was invoked 0.086 times per frame for SCAR (YOLOv12) and 0.061 times per frame for SCAR (YOLOv11). The expected additional cost is therefore 0.56 GFLOPs per frame against a baseline cost of 33.09 GFLOPs for SCAR (YOLOv12), and 0.39 GFLOPs against 32.89 GFLOPs for SCAR (YOLOv11), corresponding to increases of 1.7% and 1.2%, respectively.

#### 3.2.5. Final SCAR Output

The final detection can be expressed mathematically as

(12) $$d_{i}^{final}=\left\{\begin{array}{c} {(c_{i},\;b}_{i}^{\prime },s_{i}^{\prime }),\;c_{i}\in W\;\\ (c_{i},\;b_{i},\;s_{i}),\;c_{i}\notin W \end{array}\right.$$

## 4. Results and Analysis

The core evaluation metrics include mAP(50–95), mAP50, mAP75, precision and recall. SCAR is applied to two baseline detectors, YOLOv11 [35] and YOLOv12 [36], to show that the refinement is not specific to a single detector.

### 4.1. Detection Accuracy

**Figure 4:**
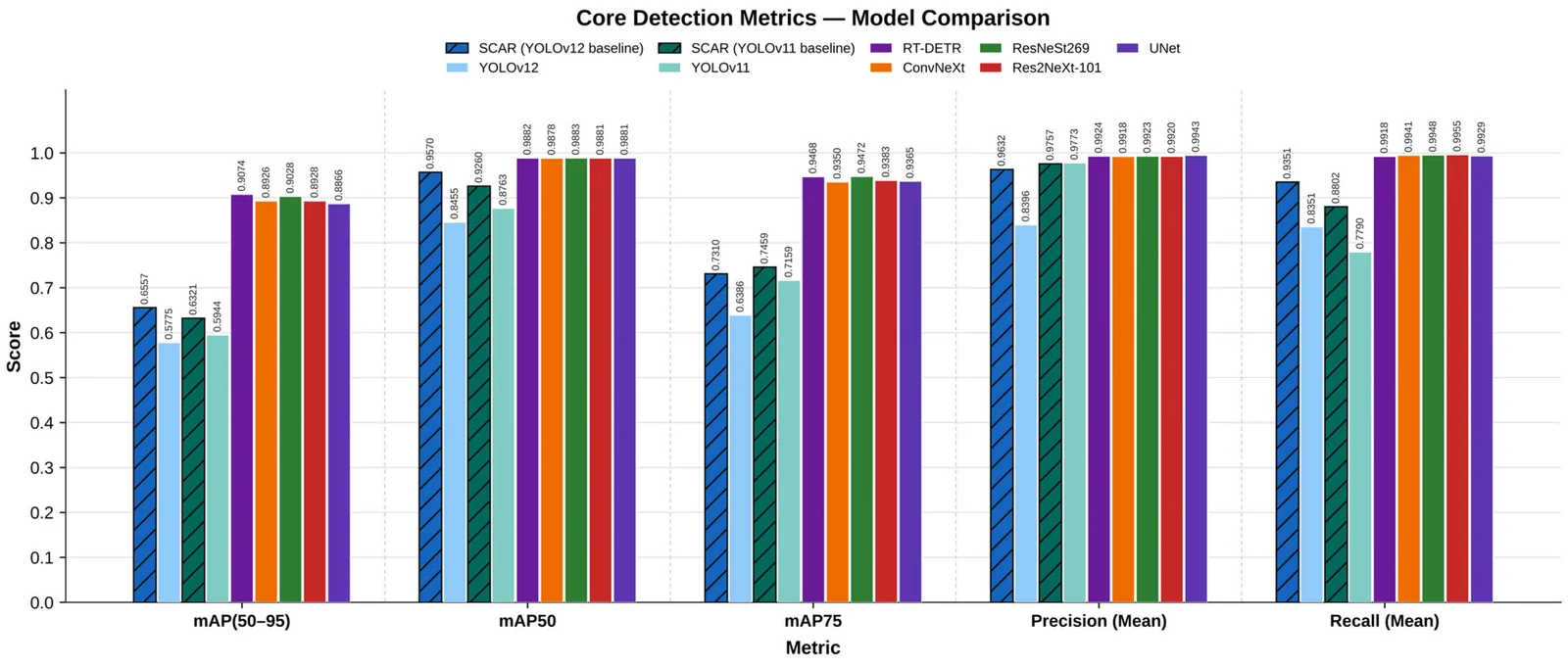
Average performance measures for Baseline and SCAR methods.

Figure 4 validates and compares SCAR’s performance against baseline detectors and state-of-the-art models. The results clearly show that SCAR consistently outperformed both baseline detectors across all metrics. mAP(50–95) measures the overall detection performance across IoU thresholds from 0.50 to 0.95. It is therefore considered the strongest performance measure, as it helps to evaluate both detection and localization quality. As depicted in Figure 4, on the YOLOv12 baseline, SCAR improves mAP(50–95) from 0.577 to 0.656, indicating greater robustness under stricter IoU thresholds. Similarly, mAP50 increases from 0.845 to 0.957. mAP75 improves from 0.639 to 0.731. This shows that SCAR targets and improves both coarse and precise localization performance. Further, the precision and recall scores rise from 0.840 to 0.963 and from 0.835 to 0.935, respectively. This shows that false-positive detections are reduced effectively, while the improved recall indicates that SCAR can successfully recover difficult microrobot instances.

The same behavior is observed with the YOLOv11 baseline, confirming that the selective refinement generalizes across detectors. As depicted in Figure 4, SCAR improves mAP(50–95) from 0.594 to 0.632 and mAP50 from 0.876 to 0.926. Recall shows the most evident gain, rising from 0.779 to 0.880, indicating that SCAR recovers microrobot instances that the baseline detector misses. The improvement is smaller than that of YOLOv12, as the YOLOv11 baseline already partially detects the hard class, leaving less to recover. However, the consistent improvement across both baselines strongly suggests the benefit comes from the refinement mechanism itself, not a single detector. Figure 4 also compares SCAR with five heavy state-of-the-art models, namely ConvNeXt [37], ResNeSt269 [38], Res2NeXt-101 [39], UNet [40] and RT-DETR [41]. These models achieve higher mAP(50–95) values ranging from 0.887 to 0.907. However, this accuracy comes at a high computational cost, which we analyze in Section 4.4. The findings in Figure 4 validate the effectiveness of SCAR in improving microrobot detection accuracy while maintaining a lightweight detection pipeline.

**Figure 5:**
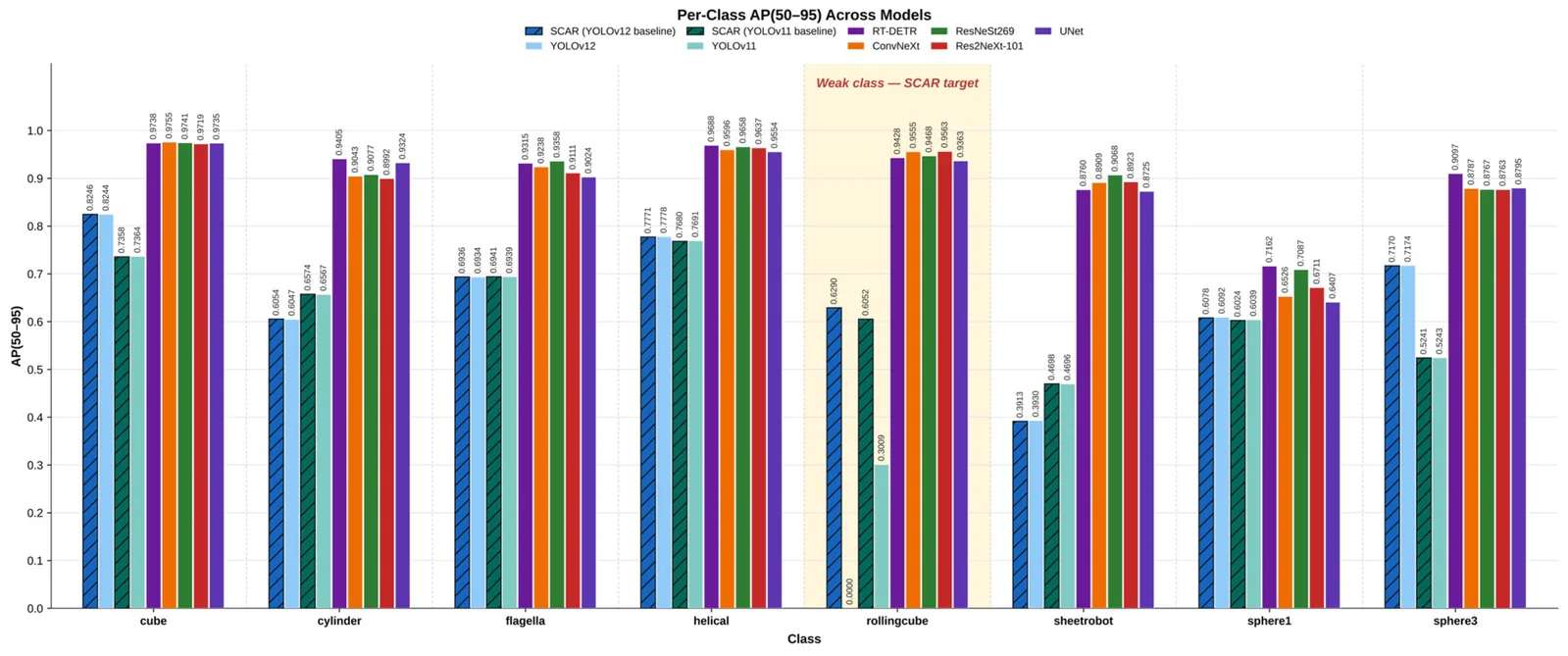
Microrobot (class-wise) detection results for all detectors compared with SCAR.

Figure 5 presents per-class detection performance and provides deeper insight into where SCAR improves detection. The baseline detectors perform well on easy-to-detect microrobot classes such as cube, cylinder, flagella, helical, sphere1, and sphere3, achieving high AP across all of them. However, the YOLOv12 baseline failed on the rollingcube class, achieving an AP(50–95) of 0.000, and the YOLOv11 baseline also performed poorly on the same class, with an AP(50–95) of 0.301 and a recall of only 0.003. As shown in Figure 2, rollingcube is a compact and localized structure with low contrast against the background, which makes it easily obscured by speckle noise and weak contrast. This is exactly where baseline detectors struggle. However, due to selective refinement, SCAR excels at this hard-to-detect class. As depicted in Figure 5, SCAR increases the rollingcube AP(50–95) from 0.000 to 0.629 on YOLOv12 and from 0.301 to 0.605 on YOLOv11, while its recall improves from 0.000 to 0.802 and from 0.003 to 0.814, respectively. It shows that SCAR recovers rollingcube instances that the baseline detectors could not detect.

Further, SCAR preserves the performance of the remaining classes. As depicted in Figure 5, the AP(50–95) of the easy-to-detect classes remains the same before and after refinement, and the sheetrobot class is also kept unchanged at 0.391 on YOLOv12 and 0.470 on YOLOv11 because selective gating refines only the diagnostically difficult class and does not disturb reliable detections. Therefore, SCAR improves the weak class without causing regression on other classes, resulting in overall improved detection performance.

The comparison is further extended to a recent transformer-based detector. RT-DETR is a real-time hybrid detector that combines a convolutional backbone with a transformer encoder–decoder, and we train it under the same protocol as the other models, using the same training split, number of epochs, and input resolution. Table 3 presents the results. RT-DETR achieves an mAP(50–95) of 0.9074, which is the highest accuracy among all evaluated models and is comparable to the heavy convolutional detectors. However, it requires 32.82 M parameters against 5.14 M for SCAR, and its throughput is 34.59 FPS against 105.14 FPS for SCAR on the same GPU. This confirms that RT-DETR belongs to the same computational regime as heavy models rather than the lightweight regime SCAR targets.

### 4.2. Statistical Reliability

To confirm that the reported improvement is reliable and not an artifact of a single training run, we repeated the experiments with five different random seeds. In SCAR, the baseline detector is a fixed trained checkpoint and its inference is deterministic, so the training of the micro-refiner is the only stochastic component of the method. Therefore, we retrained the refiner five times for each baseline, while keeping the patch construction, context expansion factor, and evaluation protocol identical.

Table 4 presents the results. On the YOLOv12 baseline, SCAR achieves a mean mAP(50–95) of 0.6548 with a standard deviation of 0.0015 and a 95% confidence interval of [0.6530, 0.6566]. On the YOLOv11 baseline, the mean is 0.6331 with a standard deviation of 0.0009 and a 95% confidence interval of [0.6320, 0.6342]. It is clearly shown that the improvement over the baselines, which are 0.0773 and 0.0387, respectively, is more than fifteen times larger than the width of the confidence interval. Therefore, the reported SCAR gain is statistically reliable and reproducible across independent training runs.

### 4.3. Frames per Second

**Figure 6:**
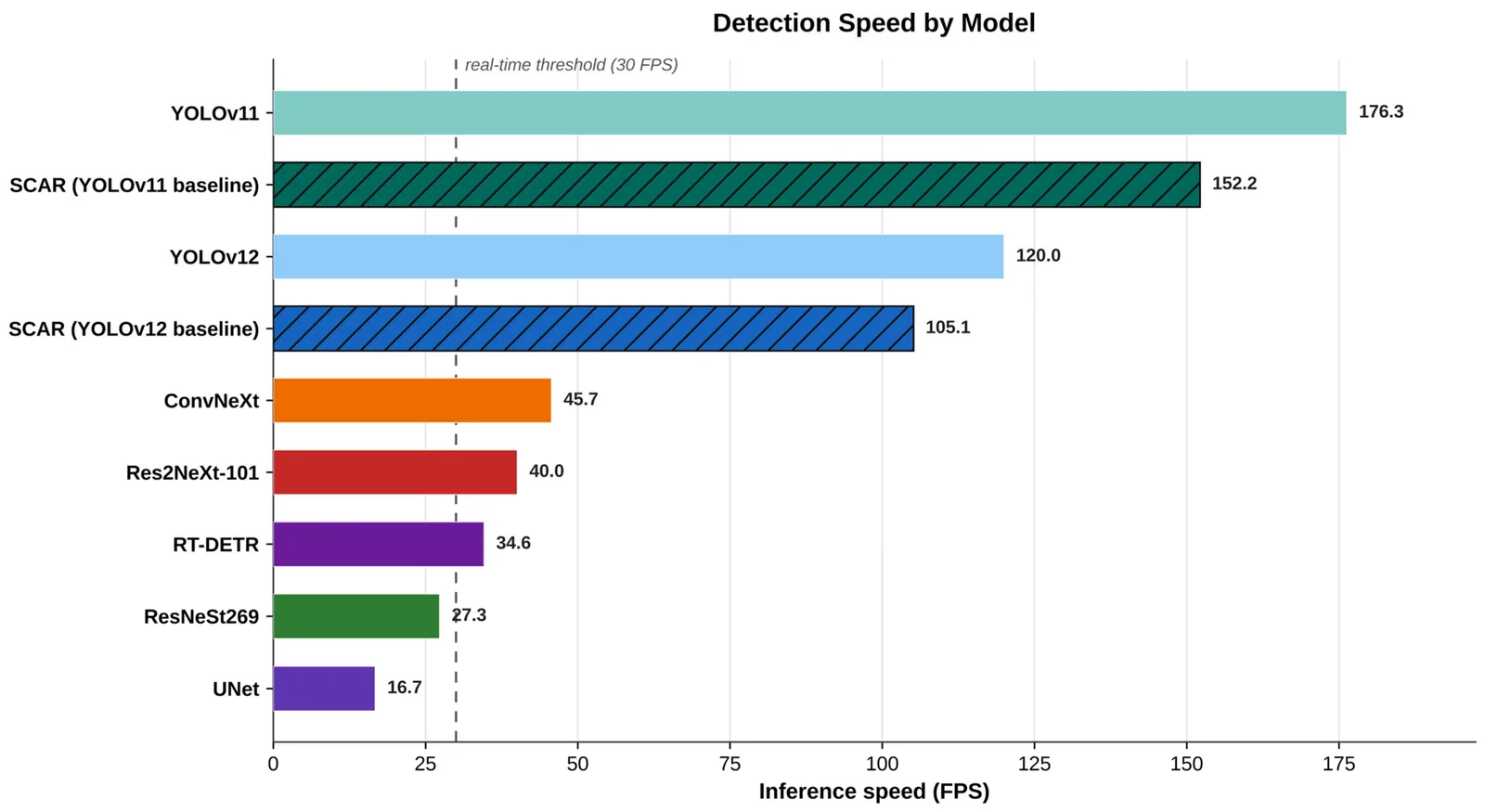
Detection speed (FPS) of SCAR vs other detectors.

Figure 6 presents the detection speeds of all models in FPS. The selective refinement mechanism of SCAR incurs a small additional computational cost for detection compared to the baseline detectors. As depicted in Figure 6, SCAR achieves nearly the same detection FPS as the baseline detectors despite fusing an additional refinement stage. On the YOLOv12 baseline, detection FPS decreases from 120.0 to 105.1, and on the YOLOv11 baseline, it decreases from 176.3 to 152.2. These small differences show that the refinement stage adds only minor computational overhead and therefore does not affect real-time clinical deployment of SCAR.

In contrast, the state-of-the-art models operate at very low detection speeds. As depicted in Figure 6, ConvNeXt, ResNeSt269, Res2NeXt-101, and UNet run at 45.7, 27.3, 40.0, and 16.7 FPS, respectively, with ResNeSt269 and UNet already below the real-time detection threshold of 30 FPS even on this high-end GPU. Although these models achieve higher accuracy, as shown in Figure 4, their limited detection speed leaves little margin for real-time microrobot tracking, as further analyzed in Section 4.4. SCAR, on the other hand, operates well above the real-time threshold while still improving the baseline’s detection accuracy. This balance between detection accuracy and high detection FPS makes SCAR suitable for practical and deployable ultrasound-based microrobot detection applications.

**Figure 7:**
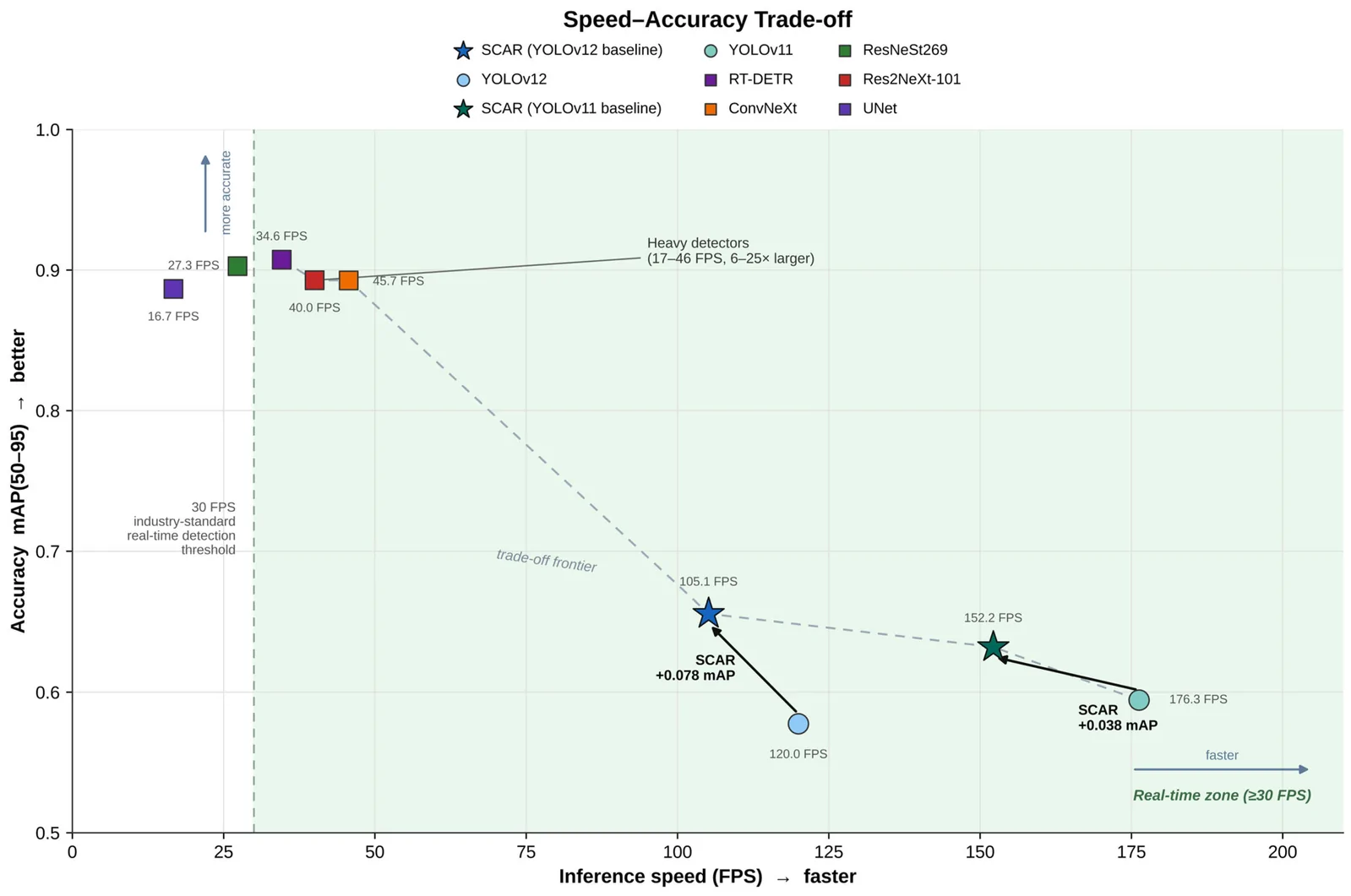
Trade-offs of higher detection accuracy vs detection speed (FPS).

Figure 7 presents the speed–accuracy trade-off for all models, with detection speed (FPS) plotted against mAP(50–95). This visualization clearly shows the position of SCAR, which is relative to both the baseline detectors and the heavy state-of-the-art models. As depicted in Figure 7, the heavy models, namely RT-DETR, ConvNeXt, ResNeSt269, Res2NeXt-101, and UNet, are placed in the upper-left region, indicating high accuracy at limited speeds of 16.7 to 45.7 FPS. ResNeSt269 and UNet fall below the 30 FPS real-time detection threshold, and the remaining heavy models sit only marginally above it, despite all being 6 to 25 times larger than SCAR. In contrast, both SCAR variants are placed in the real-time zone, well to the right of the 30 FPS threshold. As shown in Figure 7, SCAR improves the accuracy of the baseline detectors while maintaining nearly the same detection speed. On the YOLOv12 baseline, SCAR increases the mAP(50–95) by 0.078, and on the YOLOv11 baseline, it increases it by 0.038, with both moving vertically upward from their baseline without any noticeable shift in speed. This shows that SCAR pushes the baseline detectors closer to the accuracy of heavy models while still operating at real-time detection speed. Therefore, Figure 7 confirms that SCAR offers the most practical balance between detection accuracy and detection speed, making it the most deployable choice for ultrasound-based microrobot detection.

### 4.4. Computational Efficiency

**Figure 8:**
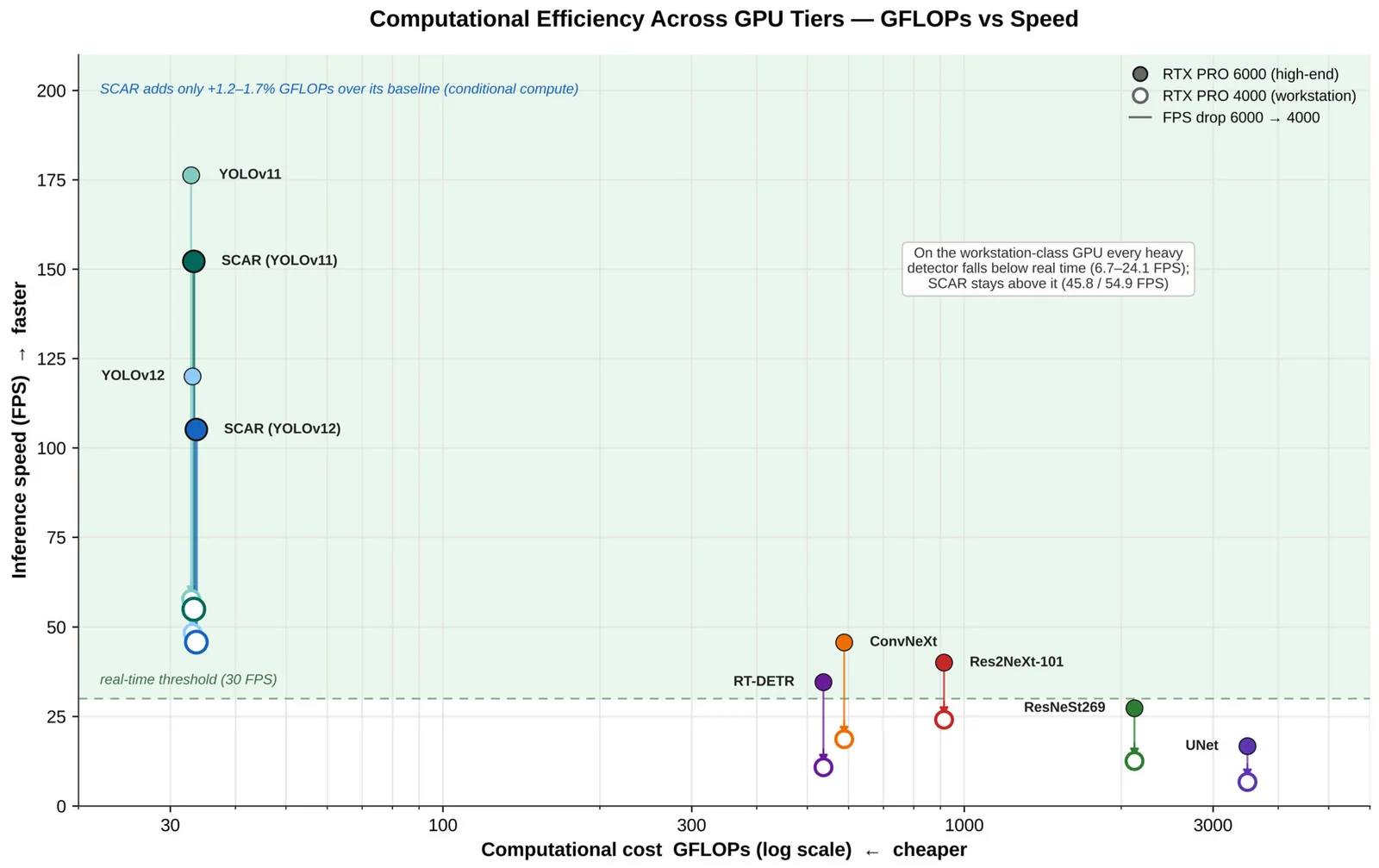
Computational efficiency across GPU tiers: hardware-independent GFLOPs against detection speed on the high-end RTX PRO 6000 and the workstation-class RTX PRO 4000.

To assess real-time deployment beyond a single high-end GPU, Table 5 reports the full computational profile of all models, and Figure 8 shows the effect of GPU tier on real-time capability. GFLOPs are hardware-independent and measured once for all models with a uniform counter at the native 1088 × 1920 input resolution, while latency and FPS are reported on two GPUs: the high-end RTX PRO 6000 and the workstation-class RTX PRO 4000, where the latter represents practical clinical deployment hardware. As stated in Table 5, SCAR adds only 0.56 and 0.39 GFLOPs per frame over the YOLOv12 and YOLOv11 baselines, corresponding to increases of only 1.7% and 1.2%. This confirms that the refiner is invoked only when needed. In contrast, the heavy detectors require 16.0 to 103.8 times more GFLOPs than SCAR, with RT-DETR at 536.83 GFLOPs and UNet at 3491.67 GFLOPs against 33.65 GFLOPs for SCAR (YOLOv12). This computational gap directly determines deployability on modest hardware. As shown in Figure 8, on the workstation-class GPU, every heavy model, including RT-DETR, falls below the real-time threshold, dropping to 6.7–24.1 FPS, whereas SCAR exceeds the real-time threshold at 45.8 and 54.9 FPS. Therefore, SCAR is the only accuracy-improving method evaluated here that retains real-time detection across both GPU tiers, which makes it flexible and suitable for practical clinical settings where high-end GPUs are not available.

## 5. Ablation Study

**Figure 9:**
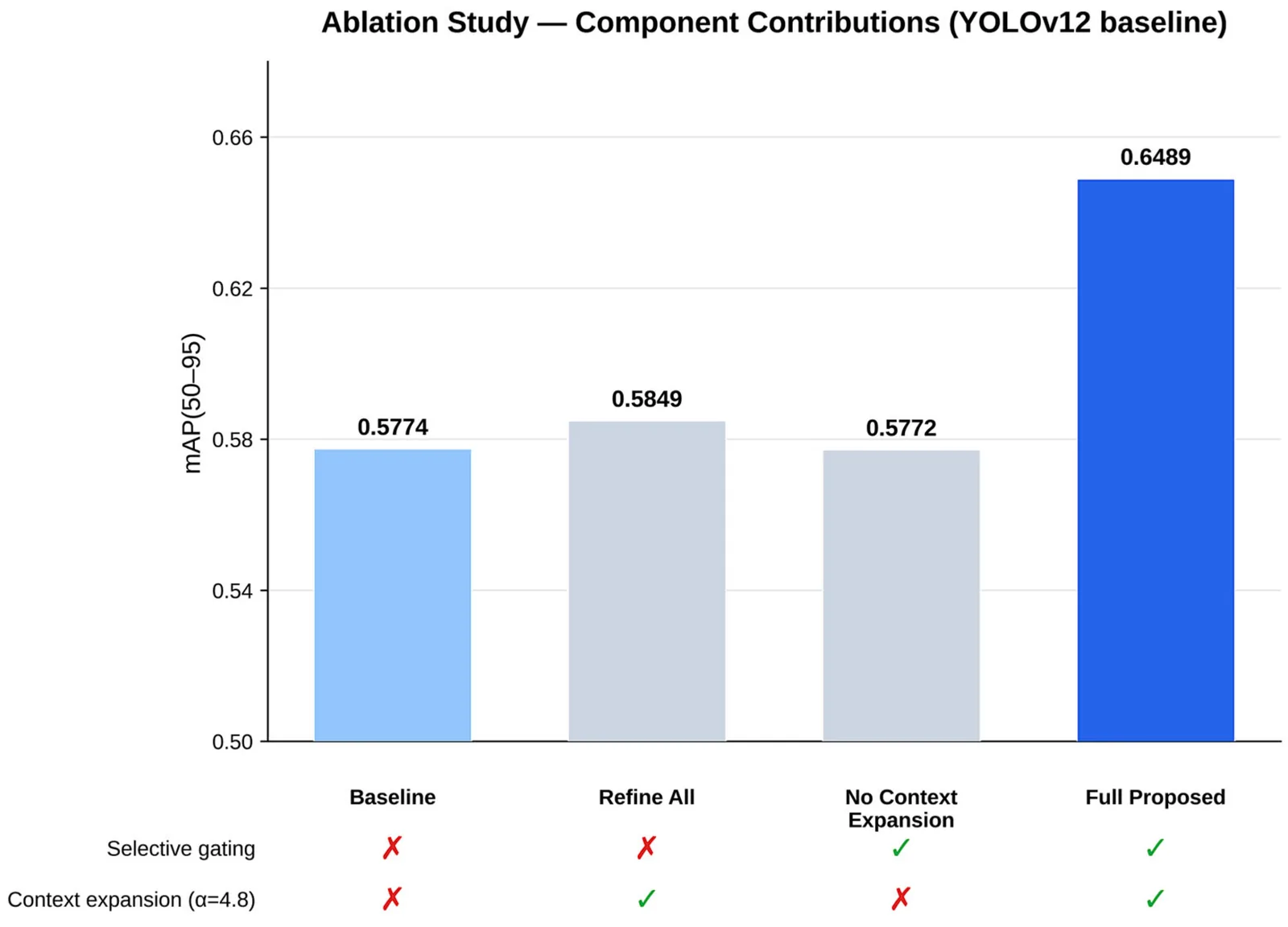
Component-wise performance of SCAR.

To analyze the contribution of each component in SCAR, we perform an ablation study in this section. The goal of this section is to determine how each component of SCAR contributes to the improvement in detection. Specifically, the ablation analyzes the effects of its two key components: selective gating and context expansion. We used four configurations for ablation purposes, as depicted in Figure 9, which are (1) the baseline YOLOv12, (2) SCAR with refinement applied to all classes without selective gating, (3) SCAR without context expansion, and (4) the full proposed SCAR method. The selective gating component tests whether refinement should be applied only to the diagnostically difficult-to-detect microrobot class or to all detections. This is important, as we do not want to disturb the reliable detections and increase computational costs. The context expansion component assesses whether the surrounding visual information is included in the local crop, which matters because, in US images, the small microrobot can be obscured by speckle noise and weak contrast. The surrounding context helps the refiner confirm the detection. We used the refiner checkpoint from our earlier experiments for this ablation, whereas Section 4 reports the updated runs. Therefore, there is a small difference in the absolute values, and this difference comes only from the checkpoint and not from the method. However, we compare all four configurations under identical settings. This shows that the relative contribution of each component is not affected.

Figure 9 presents the ablation results. The baseline YOLOv12 achieves an mAP(50–95) of 0.5774. When refinement is applied to all classes without selective gating, mAP(50–95) reaches only 0.5849, which is almost the same as the baseline. Similarly, when context expansion is removed and the refiner works on the tight crop alone, mAP(50–95) drops to 0.5772, again almost identical to the baseline. It shows that neither component, on its own, can improve detection performance. However, when both components are combined in the full proposed SCAR, the mAP(50–95) rises to 0.6489. This clearly shows that SCAR’s improvement is not due to any single component, but to the combined effect of selective gating and context expansion.

**Figure 10:**
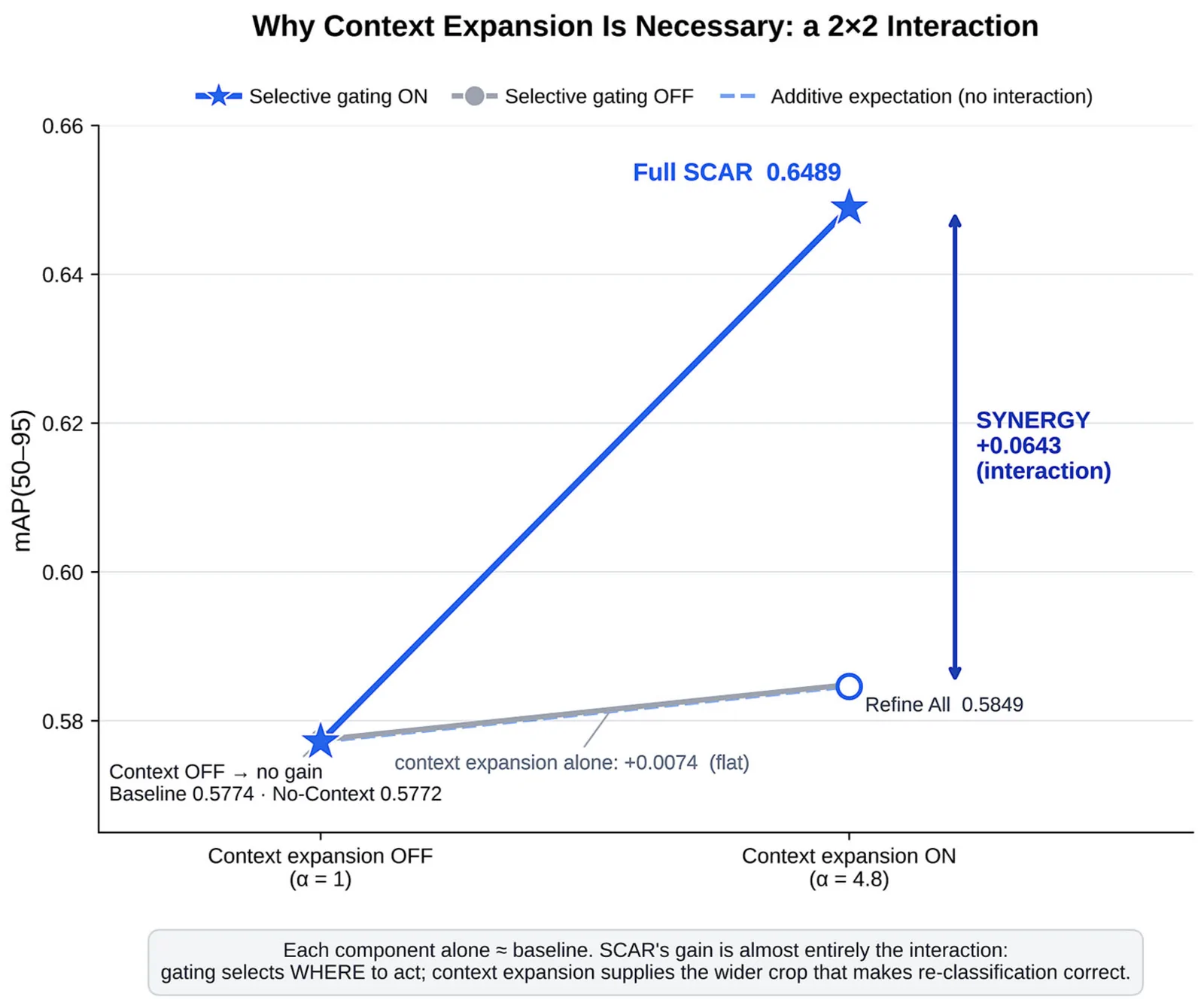
Interaction analysis of selective gating and context expansion on the YOLOv12 baseline.

To further understand why the two components must work together, we present the interaction analysis in Figure 10. As depicted in Figure 10, when context expansion is turned off (α = 1), both configurations remain near the baseline, with mAP(50–95) values of 0.5774 and 0.5772. Similarly, when selective gating is turned off and refinement is applied to all classes, the context expansion alone provides only a marginal gain of 0.0074, which is almost flat. It shows that each component, when used alone, behaves close to the baseline and provides no meaningful improvement. However, when selective gating is enabled alongside context expansion, mAP(50–95) jumps to 0.6489. As depicted in Figure 10, this gain of 0.0643 is an interaction effect, and not the simple addition of the two components.

This interaction explains the working mechanism of SCAR. Selective gating decides where to apply refinement by focusing only on the difficult-to-detect class. At the same time, context expansion provides a wider crop that includes surrounding visual information, which helps the microrobot reclassification. Therefore, gating alone cannot help if the crop is too tight to recognize the microrobot, and context expansion alone cannot help if it is applied to all detections and disturbs reliable ones. As shown in Figure 8 and Figure 9, the two components are complementary, and detection improvement for SCAR comes from their combined effect. This confirms that both selective gating and context expansion are necessary components of the proposed method.

## 6. Conclusions

Microrobots have the potential to transform how we diagnose and treat diseases. They are engineering innovations that fit into very small spaces and can navigate easily inside the human body, where traditional medical devices and equipment struggle to reach. From an operational perspective, a critical aspect is detecting microrobots inside the human body in real time to enable streamlined control and navigation. In this paper, we introduced the SCAR method, which improved the accuracy of hard-to-detect microrobot classes while maintaining real-time detection FPS. The improvement is also consistent across two YOLO baselines, showing that the refinement is not specific to a single detector. It fuses a baseline detector with a selective refinement mechanism. A limitation of SCAR is its localized selective refinement. As a result, it is highly accurate for compact microrobot structures such as the rolling cube. However, since SCAR selectively refines only the compact weak class, globally distributed structures, such as the sheetrobot, remain unchanged and are not improved by the refinement. A further limitation concerns the selection stage itself: it runs offline and is fixed at deployment, so adapting to a shifted class distribution requires re-profiling on labeled data from the new domain. As this is a single automated pass over the training split, it can be repeated periodically, but SCAR does not update its selection online. SCAR is a mechanism rather than a dataset-specific model. It identifies a recall-limited class from the error statistics of the baseline and re-inspects candidate regions locally. Nothing in this mechanism is tied to the current dataset, and its criteria are percentile-based rather than absolute, so the procedure can be re-executed on any detector and dataset. It is therefore expected to transfer to other settings that exhibit the same failure mode the baseline misses rather than over-detects. Empirical confirmation on additional US datasets remains future work. The present evaluation is confined to a single publicly available benchmark, and several conditions encountered in practice therefore remain untested. Different ultrasound devices differ in center frequency, beamforming, dynamic range, and post-processing, all of which alter the speckle statistics on which the selection criterion of Section 3.2.2 operates; the criterion is percentile-based and would re-derive its own thresholds on new data, but the resulting hard-to-detect class may differ from the one identified here. Varying image quality, arising from depth, gain, and probe placement, affects the Weber contrast used in the hard-regime filter of Equation (10), and severe degradation would enlarge the candidate set and increase the refiner invocation rate reported in Table 2. The refiner does not cover unseen microrobot geometries, as it is trained as a specialist on a single morphology; a new geometry would require re-profiling and retraining the refinement stage, although this is a single automated pass and does not affect the baseline detector. Finally, domain shift in clinical environments, including tissue heterogeneity, motion artifacts, and acoustic shadowing absent from phantom acquisitions, may introduce failure modes that are precision-limited rather than recall-limited; SCAR is explicitly gated against such classes and would therefore leave them unrefined rather than degrade them, although it would also provide no benefit in those cases. As a future research direction, we aim to record and develop our own datasets and further enhance SCAR to make it a more general method with local-to-global contextual refinement mechanisms.

## Figures and Tables

**Table 1:** Class-aware selection statistics for both baselines. The severity score ranks miss-driven failures; the gate of Equation (5) retains only classes whose miss rate exceeds their false-alarm rate. Statistics are computed offline on the training split at IoU 0.5; miss rate is per class, false-alarm rate is per image, so it may exceed 1.

Class	Score (v12)	Miss (v12)	False Alarm (v12)	Score (v11)	Miss (v11)	False Alarm (v11)
**rollingcube**	**0.472**	**0.248**	**0.023**	**0.446**	**0.211**	**0.006**
sheetrobot	0.325	0.045	0.850	0.317	0.033	1.137
sphere1	0.304	0.008	0.761	0.306	0.011	0.461
sphere3	0.301	0.007	0.212	0.303	0.010	0.153
flagella	0.297	0.012	0.123	0.293	0.006	0.153
cylinder	0.291	0.003	0.192	0.290	0.002	0.297
helical	0.284	0.006	0.234	0.283	0.005	0.294
cube	0.268	0.015	0.125	0.260	0.004	0.384

**Table 2:** Micro-refiner specifications, training configuration, and refinement cost.

Item	SCAR (YOLOv12)	SCAR (YOLOv11)
Refiner initialization	YOLOv12n baseline	YOLOv11n baseline
Output classes	1 (hard-to-detect class)	1 (hard-to-detect class)
Layers	465	320
Parameters (M)	2.568	2.590
GFLOPs per crop	7.46	6.50
Training resolution	320 × 320	320 × 320
Epochs/batch size	30/128	30/128
Precision/seed	Mixed precision/42	Mixed precision/42
Baseline parameters (M)	2.570	2.591
Baseline GFLOPs per frame	33.09	32.89
Refiner invocations per frame	0.086	0.061
Added GFLOPs per frame	0.56	0.39
Added computation over baseline	1.7%	1.2%

**Table 3:** Detection accuracy, model size, and throughput of all evaluated models.

Model	mAP(50–95)	Params (M)	FPS (RTX PRO 6000)	FPS (RTX PRO 4000)
YOLOv11	0.5944	2.59	176.27	57.74
SCAR (YOLOv11)	0.6321	5.18	152.18	54.89
**YOLOv12**	**0.5775**	**2.57**	**119.98**	**48.35**
SCAR (YOLOv12)	0.6557	5.14	105.14	45.75
**RT-DETR**	**0.9074**	**32.82**	**34.59**	**10.83**
ConvNeXt	0.8926	45.08	45.66	18.66
Res2NeXt-101	0.8928	61.04	40.04	24.10
ResNeSt269	0.9028	126.76	27.30	12.55
UNet	0.8866	31.04	16.71	6.70

**Table 4:** Variability of SCAR over five independent refiner training runs per baseline.

Baseline	Runs	Mean mAP(50–95)	SD	95% CI	Mean AP(50–95) Hard Class	SD
YOLOv12	5	0.6548	0.0015	[0.6530, 0.6566]	0.6284	0.0100
YOLOv11	5	0.6331	0.0009	[0.6320, 0.6342]	0.6130	0.0070

**Table 5:** Computational efficiency and real-time deployment capability of all evaluated models. Real-time is defined as ≥30 FPS.

Model	Params (M)	GFLOPs	Peak Mem (GB)	Latency (ms)/FPS (RTX PRO 6000)	Latency (ms)/FPS (RTX PRO 4000)	Real-Time (6000/4000)
YOLOv11	2.59	32.89	0.18	5.67/176.3	17.32/57.7	✓/✓
SCAR (YOLOv11)	5.18	33.28	0.18	6.57/152.2	18.22/54.9	✓/✓
YOLOv12	2.57	33.09	0.39	8.34/120.0	20.68/48.4	✓/✓
SCAR (YOLOv12)	5.14	33.65	0.39	9.51/105.1	21.86/45.8	✓/✓
RT-DETR	32.82	536.83	1.26	28.91/34.6	92.31/10.8	✓/✗
ConvNeXt	45.08	588.23	0.77	21.90/45.7	53.59/18.7	✓/✗
Res2NeXt-101	61.04	914.32	0.81	24.97/40.0	41.49/24.1	✓/✗
ResNeSt269	126.76	2120.40	1.07	36.63/27.3	79.70/12.6	✗/✗
UNet	31.04	3491.67	5.20	59.85/16.7	149.29/6.7	✗/✗

## Data Availability

The dataset used in this study is the publicly archived USMicroMagSet dataset [8]. It is available at IEEE DataPort: https://dx.doi.org/10.21227/1dsz-da61. It needs an IEEE DataPort subscription.
